# Borderline personality disorder and substance use disorders: an updated review

**DOI:** 10.1186/s40479-018-0093-9

**Published:** 2018-09-19

**Authors:** Timothy J. Trull, Lindsey K. Freeman, Tayler J. Vebares, Alexandria M. Choate, Ashley C. Helle, Andrea M. Wycoff

**Affiliations:** Department of Psychological Sciences, University of Missouri-Columbia, 210 McAlester Hall, Columbia, MO 65211 USA

**Keywords:** Borderline personality disorder, Substance use disorder, Alcohol use disorder, Comorbidity

## Abstract

For decades, clinicians and researchers have recognized that borderline personality disorder (BPD) and substance use disorders (SUDs) are often diagnosed within the same person (e.g., (Gunderson JG. Borderline personality disorder: A clinical guide. Washington, D.C.: American Psychiatric Press, 2001; Leichsenring et al., Lancet 377:74-84, 2011; Paris J. Borderline personality disorder: A multidimensional approach. American Psychiatric Pub, 1994; Trull et al., Clin Psychol Rev 20:235-53, 2000)). Previously, we documented the extent of this co-occurrence and offered a number of methodological and theoretical explanations for the co-occurrence (Trull et al., Clin Psychol Rev 20:235-53, 2000). Here, we provide an updated review of the literature on the co-occurrence between borderline personality disorder (BPD) and substance use disorders (SUDs) from 70 studies published from 2000 to 2017, and we compare the co-occurrence of these disorders to that documented by a previous review of 36 studies over 15 years ago (Trull et al., Clin Psychol Rev 20:235-53, 2000).

## Background

For decades, clinicians and researchers have recognized that borderline personality disorder (BPD) and substance use disorders (SUDs) are often diagnosed within the same person (e.g., [1–4]). Previously, we documented the extent of this co-occurrence and offered a number of methodological and theoretical explanations for the co-occurrence [4]. In this article, we provide an update on this co-occurrence by reviewing studies published between 2000 and 2017, inclusive, and we compare the co-occurrence rates between BPD and SUDs with our previous review. First, we briefly introduce the distinction between co-occurrence and comorbidity. Next, we provide some background and context on BPD symptoms and we highlight the conceptual and potential etiological overlap of SUDs and BPD. Third, we review and compare the data on the rates of co-occurrence between BPD and SUDs from the present and a previous review [4]. Finally, we discuss the conceptual and clinical implications of this co-occurrence to facilitate future research and treatment.

### The issue of co-occurrence and comorbidity

Psychiatric diagnostic comorbidity is a broad and complex issue, referring to both the co-occurrence of disorders within the same person and the covariation of disorders in a population [5]. Further, two distinct diseases or clinical disorders diagnosed in the same person represents “true” diagnostic comorbidity [5, 6]. Establishing true comorbidity among syndromes within psychiatry is challenging given the relatively limited etiological information known, compared to many other conditions which are known to be distinct, and is easily influenced by diagnostic classification systems. Therefore, we focus our review on “co-occurrence,” or two syndromes existing (i.e., overlapping) within the same individual at the same time, without assuming associations at the etiological level. BPD-SUD co-occurrence rates can still provide some clues as to potential shared and distinct etiology, traits, and course.

### Borderline personality disorder

Borderline personality disorder (BPD), a severe personality disorder that develops by early adulthood, is characterized by emotion dysregulation, impulsive acts, disturbed interpersonal relationships, and suicidal and self-harm behaviors [7]. BPD is the most commonly diagnosed personality disorder in both inpatient and outpatient settings [2, 8], and recent estimates suggest that BPD is relatively prevalent in nonclinical populations as well (range 2–3%) [9–11].

Although BPD is presented as a categorical disorder (i.e., present versus absent) in the DSM-5 [7], the evidence for dimensional approaches to pathological personality traits, and psychopathology more broadly, have a robust evidence base [12]. There are significant limitations with categorizing BPD, including heterogeneity within the categories, arbitrary cut-points, and high diagnostic co-occurrence [13, 14]. Dimensional approaches are consistent with the current state of classification research in the field, and this is also true for BPD. For instance, BPD can be conceptualized as maladaptive variants of general personality traits from the Five Factor Model, primarily represented by high neuroticism, antagonism, and disinhibition [15]. This is largely consistent with the DSM Alternative Model (DSM-AM) representation of BPD [7]. However, given that the studies in this updated review utilized the categorical classification of BPD as is currently retained in DSM-5, we will focus on the categorical diagnoses of BPD (and SUDs). Nevertheless, we do discuss trait-based, dimensions that may be relevant to an understanding of the co-occurrence and comorbidity of BPD and SUDs.

Disorders with the highest rates of co-occurrence with BPD are mood, anxiety, substance use, and non-BPD personality disorders [2, 8, 10]. Considering both personality disorder and non-personality disorder co-occurrence, it appears that very few patients with a BPD diagnosis fail to meet criteria for another psychiatric diagnosis. These findings are consistent with the view that BPD represents a level of personality organization/dysfunction that cuts across existing diagnostic categories [16, 17]. Not surprisingly, substantial levels of impairment are associated with BPD; individuals diagnosed with BPD are prone to attempt suicide, seek and utilize health care services, and report significant levels of impairment in personal, role, and social functioning [1–3, 10].

### Co-occurrence with substance use disorders (SUDs)

As noted by Trull et al. [4], the co-occurrence of BPD and SUDs can be understood from both methodological and theoretical perspectives. First, the association between these two disorders in studies may be due to methodological artifacts. For example, chronic, excessive use of substances as well as problems due to excessive use are potential indicators of the BPD diagnosis (i.e., the BPD impulsivity criterion [7]). To address this potential artifact, researchers have examined co-occurrence independent from these shared features and established that substantial co-occurrence remains (e.g., see [18, 19]). This suggests that co-occurrence between the two disorders is not primarily a function of symptom overlap. Another potential methodological problem in assessing this co-occurrence is that many studies of substance-using samples are cross-sectional, and the active or withdrawal phases of substance use are characterized by features that resemble criteria of BPD (e.g., affective instability, interpersonal problems [7]). Thus, it is critical that assessors establish the experience of these BPD symptoms outside of any intoxication or withdrawal phase of substance use. Finally, the co-occurrence may be primarily due to a shared third variable that is etiologically relevant to both disorders (e.g., childhood trauma, family history of disinhibitory psychopathology). Therefore, it is crucial to assess individuals for relevant third variables to rule out this potential explanation. Relatedly, one disorder may be more likely to develop from the other (or vice versa) or the two disorders may reciprocally affect the maintenance of the other. Cross-sectional research designs cannot adjudicate the direction of causal influence; only longitudinal studies can address this issue.

Concerning theoretical influences on co-occurrence, both emotion dysregulation as well as impulsivity figure prominently in etiological accounts of both disorders [20]. For example, several criteria for BPD reference negative affectivity and affective instability (e.g., chronic feelings of emptiness, affective instability, anger dysregulation [7]). According to major theories of SUDs, emotion dysregulation also plays a role in the development of excessive substance use and problems related to use [20, 21]. This may be most pronounced in later stages of addiction that are characterized by withdrawal and heightened negative affect [22]. Specifically, the use of substances may be an attempt to regulate negative emotions, through a negative reinforcement process, and coping with negative affect is one of the leading motivations relevant to substance use (e.g., [23]). As for impulsivity, this major personality feature of BPD can lead to a number of negative consequences including substance abuse and dependence. Etiological theories of SUDs also implicate impulsivity, especially in the early stages of addiction, and there is evidence that those higher in impulsivity may be more likely to experience tension reduction following substance use (i.e., a pharmacological vulnerability [20, 24]). In addition to examining the co-occurrence of BPD and SUDs, the examination of underlying factors like emotion dysregulation and impulsivity that cut across these disorders can guide research in assessing shared etiology, treatment, and clinical course. With these issues in mind, we now turn to our updated review of the co-occurrence of BPD and SUDs.

## Method

### Search protocol

**Fig. 1:**
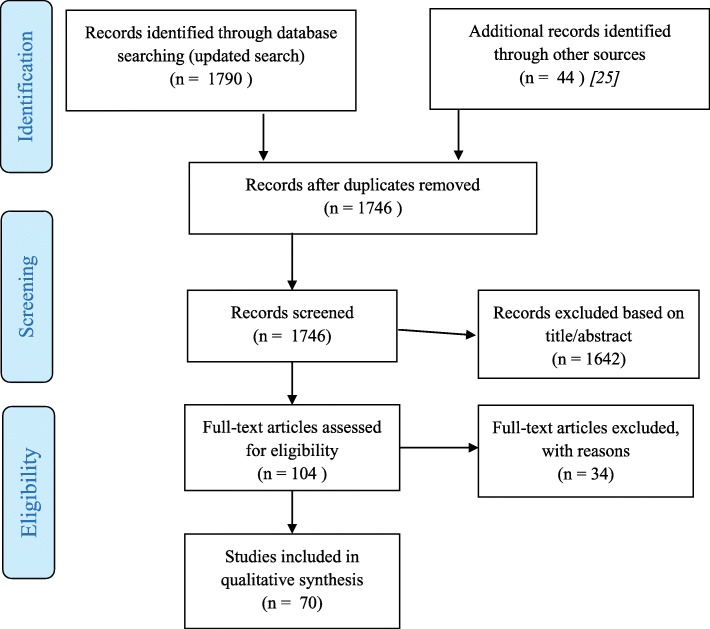
PRISMA Flow Diagram

To obtain a current estimate of the co-occurrence between SUDs and BPD we conducted a comprehensive, systematic literature search in English language journals from 2000 to 2013 (inclusive), with an updated search for articles from 2014 to 2017 (inclusive). A review of the articles from the initial search has been published [25]. For both the initial and updated search, search terms combined (“borderline personality disorder” OR BPD) with any of the following: (substance OR “substance use disorder” OR abuse OR dependence OR alcohol OR “alcohol use disorder”). The term “structured interview” was an option to refine search results. Searches queried PubMed and PsycINFO. In the updated search, Google Scholar was also queried. We reviewed titles and abstracts and evaluated articles that were returned from searches. We included the 40 studies from the initial search (2000–2013 [25]) and another 30 studies from our updated search (2014–2017). See Fig. 1 for PRISMA Flow Diagram of study selection and exclusion process [26].

### Inclusion and exclusion criteria

Inclusion criteria required each study to (a) use structured interviews using diagnostic criteria from the DSM-IV or DSM-5 to diagnose BPD, (b) use structured interviews using diagnostic criteria from the DSM-IV or DSM-5 to diagnose SUDs or sample adults in current treatment for SUDs, and (c) present sample characteristics such that co-occurrence rates between BPD and SUDs could be calculated. We excluded studies that had constraints on samples such that other comorbidities were excluded in original samples (i.e., no current substance use, no bipolar disorder, no other Axis I disorders, etc.). We also excluded studies that recruited specifically for the co-occurrence between BPD and SUDs.

In the event that multiple articles reported on the same sample of participants, we included only the article with the largest sample size. Other articles with smaller subsets of the larger sample were excluded to avoid “double counting” such data.^1^ In total, data from 70 studies are reported here in Tables 1 and 2.

**Table 1:** Prevalence of comorbid BPD in individuals with SUD

Reference	Sample	Diagnostic Instrument	N with SUD	% female	Mean Age (SD)	N with BPD (%)
Anestis, Gratz, Bagge, & Tull, 2012 [40]	Inpatient - C	SCID-I/P; DIPD-IV	176	35.8	36.12 (10.33)	53 (30.1)
Bardeen et al., 2014 [41]	Inpatient (cocaine dependence) - C	DIPD-IV	58	45	44.5 (6.6)	22 (38.0)
Bornovalova et al., 2008 [42]	Inpatient - C	SCID	76	32.9	42.2 (8.2)	24 (31.6)
Bottlender, Preuss, & Soyka, 2006 [43]	Inpatient (alcohol dependence) - C	SCID-II	237	18.1	42.0 (−-)	42 (17.9)
Dixon-Gordon, Tull, & Gratz, 2014 [44]	Residential SUD - C	SCID-I; DIPD-IV	246	36.2	35.6 (10.1)	83 (33.7)
Dunsieth et al., 2004 [45]	Residential (sex offenders without paraphilias) - C	SCID	26	0.0	39.0 (6.1)	4 (15.4)
Gonzalez, 2014 [46]	Inpatient Detoxification Unit - C	PAS	53	45.3	38.66 (8.45)	11 (21)
Gratz & Tull, 2010 [47]	Inpatient (cocaine dependence) - C	SCID-IV; DIPD-IV	61	46.0	44.45 (7.05)	24 (39)
Kopetz et al., 2014 [48]	Residential SUD - C	SCID-I; SCID-II	211	32	45 (7.05)	58 (27.49)
Krieger et al., 2016 [49]	Inpatient - C	SCID-I; SCID-II	101	30.7	40.3 (12.6)	12 (11.9)
Modestin et al., 2001 [50]	Inpatient (opioid dependence) - C	SCID-II	100	0.0	29.7 (−-)	51 (51.0)
Preuss et al., 2001 [51]	Inpatient (alcohol dependence) - C	SCID	135	20.7	41.8 (8.8)	23 (17.0)
Ross et al., 2003 [52]	Inpatient - C	SCID	100	19.0	37.1 (9.3)	39 (39.0)
Tull, Gratz, & Weiss, 2011 [53]	Inpatient - C	DIPD-IV; SCID-I/P	94	44.7	36.0 (10.07)	31 (33.0)
Vergara-Moragues, González-Saiz, Lozano, & García, 2013 [54]	Inpatient (cocaine dependence) - C	PRISM	218	8.7	–	30 (13.8)
Webber et al., 2015 [55]	Inpatient - C	SCID-II	235	53	30.06 (8.41)	120 (51.3)
Yang, Liao, Wang, Chawarski, & Hao, 2015 [56]	Inpatient (heroin dependence) - C	SCID-I; SCID-II	1002	30.04	33 (6.8)	226 (22.6)
Zikos, Gill, & Charney, 2010 [57]	Inpatient (AUDs) - C	SCID-I; SCID-II	138	33.0	44 (9.7)	19 (13.0)
Ball, 2007 [58]; Ball & Cecero, 2001 [59]	Outpatient (opioid dependence) – C	SCID-II	78	54.0 (of those with PDs)	37.4 (5.9)	23 (29.5)
Barral et al., 2017 [60]	Outpatient - C	SCID-I; SCID-II	937	23.5	37.83 (10.05)	128 (13.7)
Becker, Añez, Paris, & Grilo, 2010 [61]	Outpatient (AUD-L) - L	S-DIPD-IV	130	31.0	37.4 (10.5)	39 (30.0) - C
Casadio et al., 2014 [62]	Outpatient - C	SCID-II	320	26.3	40.9 (10.8)	48 (15)
Dammann et al., 2017 [63]	Outpatient (opioid dependence) - C	SCID-II	26	34.6	41 (6.8)	3 (11.5)
DeMarce, Lash, Parker, Burke, & Brambow, 2013 [64]	Outpatient - C	SCID-I; SCID-II	183	0.04	50.1 (8.3)	16 (8.7)
Echeburua et al., 2005 [65]	Outpatient (alcohol dependence) - C	SCID-I IPDE	30	0.0	–	0 (0.0)
Echeburua, et al., 2007 [66]	Outpatient (alcohol dependence) - C	SCID-I; IPDE	158	34.8	43.4 (−-)	8 (5.1)
Hunter-Reel, Epstein, McCrady, & Eddie, 2014 [67]	Outpatient (AUD) – C	SCID-II	102	100	45.05 (9.19)	6 (5.88)
Kidorf et al., 2015 [68]	Outpatient (opioid dependence) - C	SCID-I; SCID-II	125	53.6	39.1 (10.2)	34 (27.2)
Kok, de Haan, Wieske, de Weert, & de Jong, 2017 [69]	Outpatient - C	SIDP-IV	102	19.6	40.7 (10.8)	2 (2)
Palmer et al., 2003 [70]	Outpatient (opioid dependence) - C	SCID	107	53.0	43.1 (6.6)	40 (37.4)
Ralevski et al., 2007 [71]	Outpatient (alcohol dependence) - C	SCID	225	2.7	47.0 (−-)	68 (30.2)
Zimmerman et al., 2005 [72]	Outpatient (MIDAS) - C	SCID-I; SIDP-IV	85	–	–	15 (17.6)
Chapman & Cellucci, 2007 [73]	Incarcerated - C	TAAD; SCID-II	58 with Alcohol Dependence 73 with Drug Dependence	100	–	14 (24.1) of those with alcohol dependence 21 (28.8) of those with drug dependence
Grella et al., 2008 [74]	Incarcerated (prison-based substance abuse treatment program) - C	SCID-II	280	35.0	34.8 (−-)	37 (13.2)
Mir et al., 2015 [75]	Incarcerated - C	MINI; SCID-II	93	100	–	16 (17)
Fenton et al., 2011 [76]	Community (NESARC) - C	AUDADIS-IV	613	32.5	–	138 (22.49) of those with drug dependence
Whitbeck, Armenta, & Welch-Lazoritz, 2015 [77]	Homeless community - C	CIDI; DIPD-IV	47	100	38.9 (10.22)	25 (53.19)
Comin et al., 2016 [78]	Outpatient + Inpatient Detoxification Unit (cocaine dependence) - C	PRISM	143	18.18	34.28 (8.01)	34 (23.8)
Daigre et al., 2015 [79]	Clinical^a^ - C	EuropASI; SCID-II	512	24.1	38.8 (10.1)	66 (12.9)
Hasin et al., 2006 [80]	Inpatient & outpatient - L	PRISM-IV	285	46.0	36.3 (8.8)	56 (19.5) - L
Malik, Chand, Marimuthu, & Suman, 2017 [81]	Inpatient & outpatient (AUD) - C	MINI; SCID-II	35	100	38.51 (7.42)	6 (17)
Ross et al., 2005 [82]	Outpatient, residential, community (heroin dependence) - C	CIDI	825	35.0	29.5 (7.8)	388 (47.0)
Rubio et al., 2007 [83]	Inpatient & outpatient (alcohol dependence) - C	SCID	247	0.0	40.3 (−-)	29 (11.7)
Torrens et al., 2004 [84]	Inpatient & outpatient - C	SCID	105	31.0	33.3 (7.7)	7 (6.7)
Torrens et al., 2004 [84]	Inpatient & outpatient - C	PRISM-IV	105	31.0	33.3 (7.7)	12 (11.4)
Wapp et al., 2015 [85]	Inpatient & outpatient - C	SCID-II; MINI	1205	47.1	35.6 (9.9)	172 (14.3)

NR denotes studies in which the raw count of individuals with BPD was not provided

*C* Current diagnoses were reported. *L* Lifetime diagnoses were reported

*AUDADIS* Alcohol Use Disorder and Associated Disabilities Interview Schedule, *CIDI* Composite International Diagnostic Interview, *DIPD* Diagnostic Interview for DSM-IV Personality Disorders, *EuropASI* European Addiction Severity Index, *IPDE* International Personality Disorder Examination, *MINI* Mini-International Neuropsychiatric Interview, *PAS* Personality Assessment Schedule, *PRISM* Psychiatric Research Interview for Substance and Mental Disorders, *SCID* Structured Clinical Interview for DSM-IV Disorders, *SIDP-IV* Structured Interview for DSM-IV Personality, *TAAD* Triage Assessment for Addictive Disorders

^a^Did not specify if the sample was inpatient or outpatient

**Table 2:** Prevalence of co-morbid SUD in individuals with BPD

Reference	Sample	Diagnostic Instrument	N with BPD	% female	Mean Age (SD)	N with SUD (%)
Tadić et al., 2009 [27]	Inpatient – C, L	M-CIDI; SCID-I; SCID-II	169	70.0	32.9 (9.1)	47 (28.0) - C 115 (68.0) - L
Asnaani et al., 2007 [86]	Outpatient - C	SCID; SIDP-IV	237	72.6	31.6 (8.6)	105 (44.3)
Chen et al., 2007 [87]	Outpatient - C	SCID-I; SCID-II; IPDE	184	100	31.0 (8.0)	82 (44.6)
Comtois et al., 2003 [88]	Outpatient - L	SCID-I; PDE	29	76.0	–	25 (86.2)
Frias et al., 2017 [89]	Outpatient - C	SCID-I; SCID-II	102	91.2	35.99 (11.9)	35 (34.3)
Hidalgo-Mazzei, Walsh, Rosenstein, & Zimmerman, 2015 [90]	Outpatient (MIDAS) - L	SCID; SIDP-IV	389	71.7	32.32 (10)	AUDs = 248 (63.75) Stimulant use disorders = 11 (2.83) Cannabis use disorders = 121 (31.11) Cocaine use disorders = 61 (15.68) Hallucinogens use disorders = 17 (4.37) Opioid use disorders = 28 (7.2) Sedative use disorders = 16 (4.11) Other SUDs = 4 (1.03) Polysubstance use disorder = 38 (9.77) Any SUD = 281 (72.24)
Johnson et al., 2003 [91]	Outpatient - C	SCID-I; DIPD-IV	240	72.9	31.9 (−-)	157 (65.4)
Lane, Carpenter, Sher, & Trull, 2016 [92]	Outpatient - C	SCID-I; SCID-II	56	–	–	18 (32.1)
Maraz et al., 2016 [93]	Outpatient - C	AUDIT; SCID-II	110	54.5	34.6 (9.8)	49 (44.5) with AUD 25 (22.7) with DUD
Neacsiu et al., 2015 [94]	Outpatient – C, L	SCID-I; SCID-II	20	–	35.4 (11.6)	2 (10) - C 17 (85) - L
Perroud et al., 2016 [95]	Outpatient - L	SCID-II; DIGS	116	91.4	31.5 (9.74)	67 (62) with substance dependence 65 (60.2) with alcohol dependence
Riihimäki, Vuorilehto, & Isometsä, 2013 [96]	Outpatient - C	SCID-I/P; SCID-II	35	86.0	37.3 (13.7)	10 (29.0)
Turner et al., 2015 [97]	Outpatient - L	SCID-I; SCID-II	46^a^	91.3	31.55 (10.36)	32 (69) with any SUD 23 (50) with Alcohol Abuse or Dependence 21 (45.5) with Substance Abuse or Dependence
Welch & Linehan, 2002 [98]	Outpatient - C	SCID-I; PDE; SCID-II	122	100	31.0 (−-)	47 (35.5) with DUD
Chapman & Cellucci, 2007 [73]	Incarcerated - C	TAAD; SCID-II	50	100	–	14 (28.0) with AUD 21 (42.0) with DUD
Wetterborg, Langstrom, Andersson, & Enebrink., 2015 [99]	Probation/ Parole - C	SCID-II; MINI	11	0	33.3 (7.9)	7 (63.6) with AUD 8 (72.7) with DUD
Baschnagel et al., 2013 [100]	Community - C	SIDP-IV; CDIS; MINI	51	80.0 - of those with SUD	38.0 (9.9) - of those with SUD	35 (69.0)
Carpenter, Wood, & Trull, 2016 [101]	Community (NESARC) - L	AUDADIS-IV	1030	–	–	Alcohol use disorder = 604 (63.08); Amphetamine use disorder = 90 (9.43%); Cannabis use disorder = 281 (30.98%); Cocaine use disorder = 148 (16.73%); Hallucinogen use disorder = 75 (9.67%); Inhalant use disorder = 25 (2.93%); Opiate use disorder = 114 (13.23%); Sedative use disorder = 86 (8.43%); Tranquilizer use disorder = 69 (7.88%)
Tomko, Trull, Wood, & Sher, 2014 [10]	Community (NESARC-Revised) - L	AUDADIS-IV	1030	57.3	41.8	805 (78.2)
Widom, Czaja, & Paris, 2009 [102]	Community – C	DIPD-R; DIB-R; DIS-III-R	112	–	–	70 (62.7) with AUD 62 (55.5) with DUD
Barone, Fossati, & Guiducci, 2011 [103]	Inpatients & outpatients - C	SCID; SCID-I	140	61.0	32.39 (9.54)	40 (29.0) with SUD 40 (29.0) with AUD
Berenson et al., 2016 [104]	Outpatient & community (not all in treatment) - C	SID-P-IV	35	85.7	–	9 (25.7) with Dependence 5 (14.3) with Abuse
McCormick et al., 2007 [105]	Inpatient, outpatient, community - C	SCID-I; SIDP-IV	163	84.7	31.0 (−-)	82 (50.3) with AUD 77 (47.2) with DUD
Walter et al., 2009 [106]	Inpatients & outpatients - C	SCID-I/P; DIPD-IV	175	75.0	32.1 (7.8)	91 (52.0%) AUD 96 (54.9%) DUD

NR denotes studies in which the raw count of individuals with BPD was not provided

*C* Current SUD rates were reported. *L* Lifetime SUD rates were reported

*AUDADIS* Alcohol Use Disorder and Associated Disabilities Interview Schedule, *AUDIT* Alcohol Use Disorders Identification Test, *CIDI* Composite International Diagnostic Interview, *CDIS* Diagnostic Interview Schedule-Computerized for DSM-IV, *DIB-R* Revised Diagnostic Interview for Borderlines, *DIGS* Diagnostic Interview for Genetic Studies, *DIPD* Diagnostic Interview for DSM-IV Personality Disorders, *IPDE* International Personality Disorder Examination, *MINI* Mini-International Neuropsychiatric Interview, *PDE* Personality Disorders Examination, *SCID* Structured Clinical Interview for DSM-IV Disorders, *SIDP-IV* Structured Interview for DSM-IV Personality, *TAAD* Triage Assessment for Addictive Disorders

## Results

### Borderline personality disorder among persons with substance use disorders

Table 1 presents the rates of BPD diagnoses in those with SUDs, focusing on studies that include a SUD index sample that provides a count of individuals who were also diagnosed with co-occurring BPD. Studies are sorted and presented, in order, by **setting**: (a) solely inpatient; (b) solely outpatient; (c) forensic; (d) community; or (e) a combination of sampling methods.

#### Across settings

Overall, the co-occurrence rates between current SUDs^2^ and current BPD in these studies ranged from 0 to 53.19%. Across all studies reporting current diagnoses, a total of 10,086 individuals were sampled with SUDs (or receiving treatment for addiction), and 2228 (*22.1%*) of these individuals were also diagnosed with BPD. Please note that, throughout the rest of this article, we use the term “ % with xxx” to indicate the total number of index (co-occurring) cases divided by the total number of those receiving the other diagnosis, across all studies (i.e., a weighted average).

Ten studies specifically reported the co-occurrence rate of those with current BPD among individuals diagnosed with current AUD/alcohol dependence, ranging from 0 to 30.2% (*total n across studies =* 1495; *% with BPD = 16.99%*). Four studies sampled those with current cocaine dependence and reported a co-occurrence rate with current BPD between 13.8 and 39% (*total n across studies = 631; % with BPD = 22.03%*). Seven studies sampled those with opioid dependence (including heroin dependence) and reported a co-occurrence rate with BPD between 11.5 and 51% (*total n across studies = 2263; % with BPD = 33.80%*).

#### Within settings

Eighteen of the studies reported in Table 1 recruited exclusively from inpatient or residential treatment settings. Out of the overall sample reported in these studies with a current SUD or currently in treatment for addiction (*n* = 3267), 26.7% of individuals also met criteria for current BPD. Of the 14 studies that recruited exclusively from outpatient settings (total *n* = 2478), 15.8% also met criteria for BPD. Eight of the studies reported in Table 1 recruited participants from a combination of different settings (inpatient, outpatient, and/or community). These studies were not counted in the estimates of the inpatient and outpatient samples alone. Of the 3177 total individuals with current SUDs sampled in these combined setting studies, 23.5% were also diagnosed with BPD. Finally, three studies reported the co-occurrence between current SUDs and current BPD in forensic samples (total *n* = 446; 16.6% with BPD) and two studies reported the current co-occurrence rates in community samples (total *n* = 660; 24.7% with BPD).

### Substance use disorders among persons with borderline personality disorder

Table 2 presents the rates of SUDs in those with BPD, focusing on studies including a BPD index sample, as well as a count of individuals who were also diagnosed with concurrent SUDs. Once again, we organized studies by setting: solely inpatient, solely outpatient, forensic, community, and a combination of sampling methods.

#### Across settings

Co-occurrence rates between BPD and current SUDs reported in these studies (excluding those reporting on AUD specifically) ranged from 10 to 72.7% (% with current SUD = 45.46%). Rates between BPD and lifetime SUDs reported in these studies ranged from 45.5 to 86.2% (% with lifetime SUD = 75.28%). Eleven studies reported the co-occurrence between BPD and AUD specifically, ranging from 28 to 63.6% for current AUD diagnoses (total *n* across studies = 761; % with current AUD = 46.39%) and 50% to 63.7% for lifetime AUD diagnoses (total *n* across studies = 1581; % with lifetime AUD = 59.46%). Finally, four studies reported the rates of a current drug use disorder (DUD; i.e., an SUD other than AUD) diagnosis in those with BPD, ranging from 28.57 to 72.73% (total *n* across studies = 423; % with current DUD = 39.24%).^3^

#### Within settings

Only one study reported in Table 2 recruited exclusively from an inpatient setting [27]. Sixty eight percent of that sample met criteria for a lifetime SUD and 27.8% of the sample met criteria for a current SUD. Thirteen studies reported in Table 2 recruited from outpatient settings. Out of the overall combined sample in these studies reporting lifetime rates in outpatient settings (*n* = 600), 81.2% also met criteria for a lifetime SUD. Out of the overall combined sample in these studies reporting current diagnoses in outpatient settings (*n* = 1106), 48.8% also met criteria for a current SUD. Thirty four percent of the combined BPD sample in the two studies reporting from a forensic setting met criteria for a current AUD. Forty seven percent of the combined BPD sample in the two forensic studies met criteria for a current DUD.

Four of the studies reported in Table 2 recruited participants from a combination of different settings (inpatient, outpatient, and/or community). These studies were not counted in the estimates of the inpatient and outpatient samples alone. Three of these studies reported rates of AUD in BPD samples (combined *n* = 478; % with a current AUD = 47.5%). Four of these studies reported rates of DUDs in BPD samples (combined *n* = 513; % with a current DUD = 44.3%).

### Comparison to our previous review

Using similar search strategies and inclusion/exclusion criteria, Trull et al. [4] reviewed 36 studies published over a ten-year period, from 1987 to 1997 inclusive. Across studies that reported rates of the general category of SUD (i.e., the particular substance was not specified), 57.4% of participants with BPD received a SUD diagnosis. Across those studies that provided rates of AUD (abuse or dependence) in BPD participants, 48.8% met criteria for an alcohol use disorder. Finally, 38.0% of participants with BPD met criteria for a DUD (abuse or dependence).

The comparable rates from the present review are: (1) 45.46% of participants with BPD received a current unspecified SUD diagnosis (i.e., the particular substance was not specified), and 75.28% of participants with BPD received a lifetime unspecified SUD diagnosis; (2) 46.39% of participants with BPD received a current AUD diagnosis, and 59.46% of participants with BPD received a lifetime AUD diagnosis; and (3) 39.24% of participants with BPD met criteria for a current DUD.

Concerning co-occurrence rates of BPD diagnoses in participants with one or more SUDs (abuse or dependence), Trull et al. [4] reported that among those with unspecified SUD (i.e., single or multiple unspecified SUDs), 27.4% met diagnostic criteria for BPD. Focusing on specific, primary SUD diagnoses, 14.3% of those with alcohol abuse/dependence met criteria for BPD, 16.8% of those with cocaine abuse/dependence also received a BPD diagnosis, and 18.5% of those with opioid abuse/dependence met criteria for BPD. The comparable rates from the present review are: (1) among those with unspecified SUD, 22.1% met diagnostic criteria for BPD, (2) 16.99% of those with alcohol use disorder met criteria for BPD, (3) 22.03% of those with cocaine dependence met criteria for BPD, and (4) 33.80% of those with opioid dependence met criteria for BPD.

## Conclusions

As in our earlier review [4], our updated review of 70 studies demonstrates that BPD frequently co-occurs with SUDs, and these relations are apparent in both clinical populations and the general population. The estimates from our two reviews are fairly consistent, despite the range of populations sampled as well as the more recent time frame in the current review (i.e., 2000–2017). Approximately half of those with BPD also have at least one current SUD, most commonly AUD. Among those with a current SUD, approximately 25% also meet criteria for BPD. As for specific SUD diagnoses, those with current opioid, cocaine, and alcohol use disorder most frequently received a BPD diagnosis.

The research reviewed in this update maintained similar categorical, conceptualizations of BPD as the studies in the previous review. However, some methodological differences were present given the timeframe of the review periods. Specifically, the studies included in this updated review utilized DSM-IV or DSM-5 SUD criteria (i.e., different criteria and a different time-frame), whereas the previous review primarily included DSM-III and DSM-III-R criteria. Furthermore, the initial review primarily included studies reporting lifetime SUD diagnoses, while the current review included many studies reporting current SUD diagnoses.

As noted above, this co-occurrence can be understood in a number of ways from a theoretical perspective. Contemporary theories suggest that emotion dysregulation as well as impulsivity figure prominently in the development of both disorders [20, 21, 28, 29]. Furthermore, BPD-SUD co-occurrence may reflect common etiological processes with early expression of impaired impulse control and affective dysregulation in these conditions [20]. Concerning this purported common vulnerability, there is evidence from twin studies indicating that BPD and SUDs may share genetic influences. For example, studies have reported significant genetic correlations between *borderline personality traits* (BPTs) and substance use among adolescents and young adults [30], between BPTs and nicotine and cannabis use [31], between BPTs and alcohol, nicotine, and alcohol dependence [32], and between BPD symptoms and alcohol and cannabis use as well as alcohol and cannabis use disorders [33, 34]. Importantly, Few et al. [32] provided some evidence suggesting that the genetic correlation between BPD and SUDs may be due to shared personality traits such as neuroticism/affective instability. Findings such as these reinforce the utility of a dimensional perspective in pointing to shared, underlying etiological factors that may help to explain the observed co-occurrence of psychiatric disorders.

These findings, in concert with reviews of the phenotypic associations between BPTs, BPD and SUDs, suggest that the domains of emotion dysregulation/affective instability and of impulsivity might be targets of both etiological research on these conditions as well as treatment research that seeks to identify underlying vulnerabilities that serve to increase risk for these disorders. Unfortunately, despite the promise of current psychological treatments for BPD like *Dialectical Behavior Therapy* (DBT [35]), few randomized controlled trials have directly assessed the effects of treatment on SUD-related problems in those with BPD [36]. It is likely that to be successful in reducing substance abuse and substance-related problems, treatment may need to be modified to focus on and target specific influences in substance abuse that co-occurs with BPD [37].

The current shift towards examination and implementation of dimensional conceptualizations of psychopathology, including personality pathology, will likely aid in disentangling the relationship between BPD and SUD. Etiological research can be more targeted (i.e., common and distinct underlying traits/components), and treatments may be more trans-diagnostic in nature, rather than focused on heterogeneous categories for separate disorders.

Co-occurrence rates across studies should be considered within the context of the study methods. Methodological rigor and consistency is an important consideration when comparing prevalence rates and co-occurrence across studies and samples. Diagnostic classification can also greatly impact the variation in the “output” from all of the studies. Although we have given equal weight to study estimates without judging the methodological rigor of each, prevalence and co-occurrence rates can differ dramatically depending on how well diagnostic criteria and diagnoses are operationalized and assessed. Future studies should be very explicit in how diagnostic criteria and diagnoses are operationalized. For instance, is impairment a necessary component for judging a diagnostic criterion to be present? NESARC prevalence rates of personality disorder and substance use disorders differed significantly when criteria with impairment versus criteria without impairment were used to calculate the prevalence of personality disorder diagnoses [11, 38] . When impairment was required for criteria to be considered for the diagnosis, the prevalence rates of PDs decreased, whereas the co-occurrence of many PDs (including BPD) and SUDs increased [11].

The question of whether SUDs are a cause or consequence of BPD cannot be answered definitively by our review of the existing research. However, because common genetic, personality, and early environmental influences predate overt substance use, it seems unlikely that PDs are simply secondary to substance use disorder. The effect of SUD on PD expression appears to be one of exacerbating PD symptomatology and, in turn, contributing to chronicity. This may be a transactional process at various levels of analysis; for instance, neuroadaptations of reward systems may occur as a result of chronic distress (associated with negative affectivity), thereby influencing the development of or risk for substance use [39]. This has important treatment implications in that clinicians must keep in mind the challenges present when planning and implementing treatment for those with both SUD and PD. It may be the case that variations on existing treatments or even new treatments are needed for this co-occurring condition. Regardless, prospective studies are needed to address the specific onset and course of the relationship between syndromes, as well as the exact transactional and temporal nature of these associations.

## Footnotes

1 Carpenter, Wood, & Trull (2016) [101] and Tomko, Trull, Wood, & Sher (2014) [10] used the same sample but differed in how SUDs were reported (i.e. reporting an overall rate of SUDs vs. reporting more specific SUDs), so both studies were included.

2 Only one study reported in Table 1 recruited an index sample with lifetime (rather than current) substance use disorder (Becker, Añez, Paris, & Grilo, 2010 [61]); the rate of BPD in that sample was 30%. Similarly, one study reported the lifetime rate of BPD in the sample (Hasin et al., 2006 [80]), which was 19.5%. These two studies are not included in any of the calculations in this section.

3 Note that we did not aggregate the specific substance use disorders for the sample reported in Carpenter, Wood, and Trull (2016) [101] or the sample reported in Hidalgo-Mazzei et al. (2015) [90], since individuals may have received more than one substance use disorder diagnosis.
